# Influence of Aggregate Composition on the Properties of Recycled Concrete and Improving Performance Using Special Additives

**DOI:** 10.3390/ma18051108

**Published:** 2025-02-28

**Authors:** Kitti Banyai, Oliver Czoboly, Krisztian Menyhart, Zoltan Orban

**Affiliations:** 1 University of Pecs, Faculty of Engineering and Information Technology, Structural Diagnostics and Analyses Research Team, H-7624 Pécs, Hungary; banyai.kitti.1989@gmail.com (K.B.); czobolyo@btclabor.hu (O.C.); 2 Beton Technológia Centrum Ltd., H-1107 Budapest, Hungary; menyhartk@duna-drava.hu

**Keywords:** recycled aggregate, recycled concrete, aggregate properties, admixtures, sustainable, workability, setting time

## Abstract

The principles of the circular economy and the effective utilization of construction and demolition waste are becoming increasingly important, as evidenced by a growing body of research in this field. However, studies focusing on the waterproofing properties and setting times of recycled concrete derived from various construction and demolition waste sources remain scarce. This research investigates the characteristics of recycled aggregates from different origins and explores how these characteristics influence the properties of concrete. The study examines the effects of pre-soaking aggregates to saturation, the incorporation of water absorption-reducing additives, and the ratio of recycled aggregates to natural aggregates on the properties of both fresh and hardened concrete. Laboratory tests were conducted on crushed recycled concrete aggregates (RCA), confirming that concrete produced with recycled aggregates can meet standard requirements for compressive strength and water resistance exposure classes despite the recycled aggregates themselves not meeting the required standards. The results were used to calculate an effective water–cement ratio and establish a correlation between this ratio and compressive strength. The findings indicated that the compressive strength of mixtures approached, and in most instances exceeded, that of the reference concrete utilizing natural aggregates. Specifically, the reference concrete (REF-1) achieved a compressive strength value of 51.4 MPa after 28 days, whereas the 30% recycled mixture (REC-10), made from pure concrete demolition waste, produced a compressive strength of 62.7 MPa. The maximum water penetration depth of the REC-10 mixture was measured at 11 mm, in comparison to 15 mm for the reference mixture (REF-1). Additionally, the initial setting time of the mixtures incorporating special additives reached the 80 min threshold. The practical aspects of this research examined potential industrial applications that do not necessitate special aggregate treatments, thus maintaining the water–cement ratio within acceptable limits. This study evaluates the feasibility of utilizing recycled concrete aggregates (RCA) from construction waste to produce concrete that satisfies the standard requirements for compressive strength and water resistance. It assesses the impact of RCA on performance, provides industrial insights, and suggests potential regulatory revisions.

## 1. Introduction

The development of sustainable construction materials is a key priority in contemporary civil engineering, driven by the necessity to mitigate the environmental impact of construction activities and reduce the depletion of natural resources. Among these efforts, the reuse of construction and demolition waste (CDW) in the production of recycled aggregate concrete (RAC) has emerged as a promising approach. The fundamental objective of this research is to investigate the mechanical properties of RAC, with particular emphasis on its compressive strength, setting time, and impermeability, which are critical for ensuring its practical applicability in structural and non-structural applications.

Recycled concrete aggregates (RCA) have gained increasing attention as a sustainable alternative to natural aggregates in concrete production, aligning with the principles of the circular economy. Effective utilization of construction and demolition waste is crucial for reducing environmental impact and promoting resource efficiency. However, challenges remain in ensuring the mechanical performance of RCA-based concrete, particularly regarding its water absorption properties and setting time.

The compressive strength of RAC is a primary determinant of its structural viability, yet existing studies have demonstrated that its performance is influenced by several factors, including the quality of recycled aggregates, mix proportions, and the presence of residual cementitious materials. Furthermore, the setting time of RAC, which governs its workability and construction feasibility, varies significantly based on the composition and treatment of recycled aggregates. Additionally, the impermeability of RAC is a crucial parameter that affects its resistance to water ingress and environmental degradation, necessitating an in-depth analysis of its pore structure and permeability characteristics.

A comprehensive review of prior research has been conducted to identify existing knowledge gaps and advancements in these domains. The following tables (Table 1, Table 2 and Table 3) provide a structured summary of the studies that focus on the compressive strength, setting time, and impermeability of RAC, facilitating an understanding of the challenges and potential solutions in optimizing its performance.

The compressive strength of RCA concrete is influenced by several factors, including the RCA replacement ratio, aggregate quality, and mix design modifications. Various studies have reported both strength reductions and improvements depending on these factors.

These studies indicate that RCA concrete can achieve acceptable compressive strength if appropriate mix designs and additives are used. However, high RCA content (>50%) generally leads to significant strength reductions. The setting time of RCA concrete is critical in construction applications, particularly for ensuring proper workability and placement. RCA’s high water absorption can drastically alter setting behavior.

These results suggest that RCA concrete tends to exhibit shorter setting times without treatment. The use of water-reducing admixtures and aggregate pre-saturation can mitigate this issue and ensure adequate workability. Impermeability is a key durability factor, as RCA’s high porosity may lead to increased water absorption and permeability, affecting the long-term performance of structures.

These findings highlight that RCA concrete can achieve adequate impermeability when appropriate mix designs and treatment methods are applied. However, without modification, permeability tends to increase with RCA content, requiring careful consideration for structural applications exposed to water.

There is a growing emphasis on environmental awareness and the adoption of circular economy principles, extending beyond the framework of sustainable development [20]. This emphasis is becoming increasingly important for both businesses and institutions that deal with the construction industry and demolition waste. A vast body of literature exists on the recyclability of concrete, with numerous studies delineating the characteristics of hardened recycled concrete in comparison to conventional concrete [21,22,23,24]. Some studies describe the industrial applications of recycled concrete in its raw state, such as Fong et al. (2002) who explored large-scale production and implementation throughout construction projects in Hong Kong and concluded that recycled aggregates could be used to produce quality concrete for structural applications. However, more research and development are needed to further promote the concept of recycling and broaden the scope of applications for recycled aggregates [1,2].

Ramakrishnaiah et al. (2018) studied the factors influencing the strength characteristics of fly ash-based geopolymer concrete with the addition of different proportions of polypropylene fibers. To achieve this, an extensive study was carried out to investigate numerous properties, such as the workability, compressive strength, and flexural strength, of geopolymer concrete with varying percentages of polypropylene fibers [4]. In their study, Lim et al. (2011) dealt with the cost of producing recycled aggregates, assessing their carbon footprint, and calculating a cost–benefit analysis for their recovery. Their report describes research into the feasibility of a practical concrete mix design method aimed at overcoming the barriers that limit its application in structural concrete [3].

Tiznobaik et al. (2020) conducted two full-scale field studies that focused on large-scale production and sustainable site implementation. Their research highlights the potential of using recycled concrete aggregates (RCA) as substitutes for natural aggregates in concrete production, particularly in achieving compressive strength classes C20/25 and C30/37. Additionally, their research revealed that the compressive strength of RCAs notably increases after 28 days. In Canada, this study was the basis for updating the standards in force at the time [7]. Efforts have also been made to enhance the properties of recycled aggregates through various physical and chemical treatments [25].

Ismail and Ramli (2014) proposed a method and documented the effects of leaching cement paste from recycled aggregates using low-concentration acid solutions. They concluded that controlling the moisture content of coarse RCA after surface treatment significantly affects the properties of fresh and hardened concrete [5,6]. In their research, published in 2024, Jing Zhang et al. focused on modifying the surface area of recycled coarse aggregates and the effects of changing the replacement ratio on the workability and compressive strength of fresh concrete [8]. Similar investigations have focused on the impact of cement paste in recycled aggregates and how it affects both fresh and hardened concrete. Robalo et al. (2021) reported that recycled aggregate has high porosity and water absorption due to the presence of hardened cement paste [11].

Aili et al. (2020) reported that the microstructure of concrete made with cement-based recycled aggregates is more complex than that of normal concrete. The structure of the interface transition zone affects the ultimate strength of cement-based recycled materials because of the increased absorption of water through pores and cracks [12]. Zhang et al. (2022) attributed the lower overall efficiency of recycled aggregates than that of natural aggregates to the presence of old hardened mortar within the aggregate [26]. Furthermore, Ahmad SI (2017) investigated the water permeability of concrete made with crushed clay brick as a coarse aggregate and reported that it was significantly higher (225% to 550%) than that of concrete made with natural aggregate, with the permeability directly related to compressive strength, water absorption, and porosity [16]. Revathi et al. (2014) noted significant disparities in water absorption and strength properties between recycled and natural aggregates, mainly in the former’s high water absorption capacity and low apparent density [27].

The multifaceted analysis conducted by Maja Kępniak and Paweł Łukowski in 2023 evaluated the environmental, economic, and technical aspects of utilizing recycled sand from construction and demolition waste as a fine aggregate for mortar production [28]. Similarly, Mohammed Abed et al. (2024) developed an innovative three-step methodology for assessing the quality of coarse aggregate mixtures containing recycled concrete aggregate for use in structural concrete [29]. Despite advancements, the widespread adoption of recycled aggregates in concrete production remains hindered by concerns regarding the durability of recycled aggregate concrete (RAC). Numerous studies have scrutinized the physical and mechanical properties of RAC [30], yet challenges, particularly with respect to sulfate resistance, persist.

A literature review by Lautaro R. Santillán (2024) delved into the sulfate resistance of RAC, emphasizing the interplay between the new matrix of recycled aggregate concrete (RAC) and the old matrix of recycled aggregates (RA), underscoring the critical role of sulfate-related degradation processes [31]. According to Ju et al. (2023), the treatment of RCA with CO_2_-accelerated carbonization effectively improves the macroscopic properties of RCA [24]. A study by Badraddin et al. (2021) identified the main challenges of concrete recycling in construction projects and identified and compared different groups of challenges across different organization sizes [15].

Caroline Santana Rangel et al. (2020) investigated the freeze–thaw performance of normal and high-strength RACs produced from RCAs of diverse origins. Ten concrete mixtures were subjected to multiple freeze–thaw cycles (0, 150, and 300) to study the deterioration of the main physical and mechanical properties, compressive strength, elastic modulus, and tensile strength [32]. Furthermore, Jan Skocek et al. from Heidelberg Materials R&D undertook a comprehensive study on selectively separating industrial demolished concrete into recycled sand and coarse aggregates while also removing cement paste, followed by an assessment of their suitability for structural concrete production. The physical and mechanical properties of the coarse and fine fractions of recycled aggregate and the properties and performance of the resulting concrete, such as durability, compressive strength, chloride migration, carbonation, and freeze–thaw, were extensively investigated [17].

Sampaio, C. H. et al. (2021) characterized the properties of concretes made from both recycled fine and coarse aggregates of three different strengths: C16/20 (“normal concrete”), C50/60 (“high-performance concrete”) and C70/85 (“ultra high-performance concrete”) [10]. While several recent studies have focused on modeling the performance of recycled concrete in precast structures, the work of Fan Wang et al. (2019) stands out for its simplicity and practicality, offering an effective numerical method for such analyses [14]. Chuheng Zhong et al. (2022) investigated the suitability of initial capillary water absorption as an indicator of the water absorption capacity of recycled concrete, facilitating the development of models to predict water absorption during freeze–thaw cycles with different compositions of RCA [19].

A 2014 study by Julia García-González et al. explored the feasibility of a pre-saturation technique to solve water absorption issues in the recycled aggregates used in concrete production. The results demonstrated that, when the recycled aggregate was submerged in water for a short time, the consistency of the freshly recycled concrete improved, but a slight decrease in the compressive strength was also recorded [13]. In 2022, Haizhou Li et al. presented the mechanical properties of concrete made from 100% recycled aggregate (RAC) along with an analysis of accelerated sulfate resistance. The results show that recycled aggregate can replace natural coarse and fine aggregates. The results of the mechanical property analysis show that the compressive strength of RAC is lower than that of NAC, but the difference between the compressive strengths of 100% RAC and NAC decreases with age. The results of the accelerated sulfate resistance test revealed that the performance of 100% RAC was lower than that of NAC for the dry–wet process and sulfate attack coupling [33].

The substitution of natural aggregate with recycled aggregate (RCA) in concrete mixtures has the potential to reduce the environmental constraints associated with recycling technologies. A study by Al-Kheetan et al. (2023) aimed to improve the properties of concrete containing RCA using nano-ZnO particles. The performance of the concrete mixtures was evaluated on the basis of their physical, mechanical, and durability properties. The addition of nano-ZnO particles to concrete with RCA improved its pore structure and reduced its water absorption. In addition, nano-ZnO treatment increased the compressive strength of mixtures containing 30 m% and 50 m% RCA by 2.8% and 4%, respectively [18].

One of the primary issues in RAC production lies in the performance of the aggregates used, particularly their resistance to carbonation. While the higher porosity of recycled concrete admixtures may seem disadvantageous, it is essential to consider several factors in the analysis. Research by Carlos Pico-Cortés et al. (2023) highlights that the carbonation of RAC is influenced by several variables, with the porosity of crushed concrete and cement paste being among the most significant factors [34]. Zhong et al., in 2022, investigated the effects of nano-TiO_2_ on 12 different types of concrete mixtures at the micro- and macrolevel before and after 24 h of freeze–thaw cycles. They concluded that adding nano-TiO_2_ to recycled aggregate concrete effectively improved the durability of the concrete. The pore structure directly affects the capillary water absorption performance of recycled aggregate concrete, and the porosity correlates with capillary water absorption [35].

Yang et al. (2023) investigated the original properties of RCAs and conducted experiments on RCA with accelerated carbonation modification treatment under various curing conditions. The carbonation ratio of RCA exhibited a monotonic upward trend with an increasing carbonation duration [36].

Evangelista and de Brito (2016) conducted flexural tests on reinforced concrete beams that were made with recycled fine aggregates instead of natural aggregates. The results of these tests were compared to those of a reference beam made with conventional concrete [37]. Pan et al. (2017) reported that curing recycled fine aggregates (RFAs) under optimal conditions (ORFAs) significantly reduced the crush value, water absorption, and powder content. The researchers also attempted to increase the carbonatable compound content in demolition RFAs by pre-soaking them in calcium hydroxide (CH) to increase the effectiveness of carbonation [38].

Guo et al. (2022) came to a similar conclusion in their study on the effects of recycled fine aggregates (RFA) on concrete permeability, drying shrinkage, carbonation, chloride ion penetration, acid resistance, and freeze–thaw resistance. Their results show that the old mortar content and the quality of the recycled concrete are closely related to the durability of the finished RFA concrete [39]. 

An empirical predictive model was developed to assess the potential CO_2_ sequestration potential of recycled concrete aggregates undergoing accelerated carbonation as a function of the carbonation conditions and RCA properties. The developed model was able to predict CO_2_ uptake as a function of relative humidity, grain size, carbonation duration, and cement content of the RCAs under the specific carbonation conditions investigated [40].

Increasing the sustainability of engineered cementitious composites (ECC) with good mechanical strength and ductility can be achieved by optimizing the content of recycled powder (RP) as a binder and sand replacement. ECCs blended with RPs show promising environmental and economic benefits, as RPs can be collected from construction waste when developing ECCs [41].

Several studies have investigated the modeling of the modulus of elasticity of concrete that contains recycled aggregate using composite material models. Moment et al. (2023) developed a model to estimate the modulus of elasticity of recycled aggregate concrete using software modeling and experimental data, with an estimation accuracy of 95%. Like normal concrete, the compressive strength and modulus of elasticity of recycled aggregate concrete decrease with increasing porosity [42]. Our research aims to develop concrete that has performance characteristics that meet or surpass the strength and water resistance requirements of concrete mixtures with recycled aggregates. Additionally, we attempted to reach a comparable level of workability and initial setting time as those of concretes made from natural aggregates.

The high porosity and water absorption of crushed concrete pose significant challenges to the application of recycled aggregates in concrete technology, potentially impacting the workability and initial setting time. To promote widespread recycling, it is imperative to modify the properties of recycled aggregate or recycled concrete under standard concrete plant conditions. One common approach involves pre-soaking the recycled aggregate, in a similar way to crushed stone, to mitigate excessive water absorption. The effects of excess water on workability, compressive strength, and water resistance were investigated. To overcome the high degree of water absorption and the need to introduce additional water, several aggregate manufacturers have developed new additives, and our current research focused on the effects of these additives on the properties of fresh and hardened concrete.

The purpose of our research is to evaluate the feasibility of using RCA from construction and demolition waste to produce concrete with properties that meet or exceed the standard requirements for compressive strength and water resistance. This study investigates the effects of RCA characteristics, presoaking, admixtures, and substitution ratios on concrete performance, providing insights into their practical industrial applications and suggesting revisions to existing regulations to enable broader use of recycled aggregates. The study also highlights the unique physical and mechanical properties of recycled aggregates and emphasizes the need to thoroughly test their characteristics in specific mixtures before use.

Despite significant advancements in the utilization of recycled aggregates in concrete production, several unresolved challenges persist, warranting further investigation. One of the primary concerns is the high porosity and water absorption of recycled aggregates, which adversely affect the overall performance of RAC by increasing its water demand, reducing its workability, and potentially compromising its long-term durability. Moreover, the variability in the composition and physical properties of construction and demolition waste leads to inconsistencies in the mix design, necessitating adaptive strategies to maintain uniform mechanical performance.

A critical limitation in current RAC research is the inadequacy of setting time optimization methodologies. While pre-soaking and surface treatments of recycled aggregates have demonstrated some efficacy in mitigating premature water absorption, their effects on early-age hydration kinetics and long-term setting behavior remain ambiguous.

Another fundamental issue is the permeability of RAC, which directly impacts its resistance to aggressive environmental conditions, including freeze–thaw cycles and chloride ingress. Conventional waterproofing approaches, such as supplementary cementitious materials and pozzolanic additives, have shown the potential to enhance RAC properties. However, achieving an optimal balance between impermeability and mechanical integrity remains a key research objective, particularly for high-performance and structural applications.

This study aims to address these critical gaps by systematically evaluating the interplay between recycled aggregate composition, admixture technologies, and concrete performance parameters. Specifically, the research will:

Conduct an extensive assessment of the mechanical properties of RAC, focusing on strength development across various substitution levels and curing regimes;
Investigate the role of pre-soaking treatments and advanced admixtures in modulating setting time, ensuring consistency across different recycled aggregate sources;
Examine the permeability characteristics of RAC through water absorption tests;

By implementing these investigative frameworks, this research aspires to contribute novel insights into the sustainable and reliable utilization of recycled aggregates, paving the way for enhanced standardization and regulatory adaptations in the field of eco-friendly concrete technology.

## 2. Materials and Methods

### 2.1. Raw Materials

#### 2.1.1. Recycled Concrete Aggregates (RCA)

Recycled additives from four different sources were used in the experiments as follows:

R0. ‘reinforced pool’: reinforced concrete pool demolition material directly from the demolition site (estimated strength class: C25/30);
R1. ‘Precast’: a reclaimed byproduct of precast slab panels scrapped during pre-casting plant product testing due to geometric nonconformity (estimated strength class: C40/50);
R2. ‘Pure concrete demolition’: demolition waste from an industrial reinforced concrete frame building from an unknown location sourced from a recycling site;
R3. ‘Mixed demolition waste’: mixed demolition waste (mainly concrete waste and other brick and ceramic content) from unknown sources sourced from a recycling site.

The process involved crushing demolished concrete using a jaw crusher to achieve a fraction size of 0–150 mm, followed by waste separation using a magnetic separator. Some aggregates are a mix of crushed concrete rubble along with ceramics, wood, metal, and cohesive materials, such as clay or soil, necessitating multiple stages of manual separation and washing. The prepared raw materials were tested and were utilized in 4/8 and 8/16 fractions. Recycled aggregates were added at 0%, 15%, and 30% coarse aggregate fractions.

A uniform mixing method and sequence were applied across all of the mixtures. Both the fine and coarse fractions of the natural and recycled aggregates were subjected to saturation surface drying until they reached a constant weight in a drying oven set at 105 °C. The samples were subsequently cooled to room temperature before mixing and stored for one day in the laboratory. The particle size distribution curves of the recycled and natural aggregates exhibited similar trends for the corresponding fractions. The angular geometry and surface texture of the recycled aggregate influence the workability of fresh concrete, necessitating a higher cement paste content than that of spherical conventional aggregates.

#### 2.1.2. Natural Aggregates, Graded Sand, and Gravel

**Figure 1:**
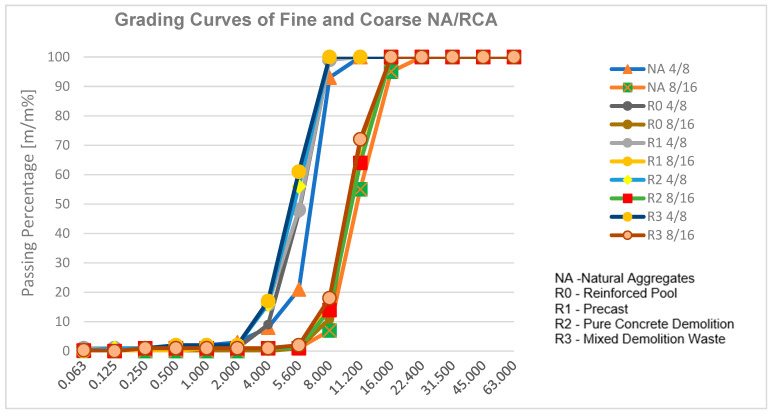
Grading curves of the fine and coarse aggregates.

The graded natural aggregates used were from the Bugyi quarry of Duna-Dráva Cement Ltd., Hungary. The aggregates were used in 0/4 mm, 4/8 mm, and 8/16 mm fractions. These aggregates are marked with NA. The grading curves for recycled and natural aggregates are depicted in Figure 1 [43].

#### 2.1.3. Cement

All of the concrete mixtures in our experiments were produced using CEM II/B-S 42.5N Portland slag cement [44] as the binder material. This cement was manufactured at the Váci cement plant of Duna-Dráva Cement Ltd., Vác, Hungary.

#### 2.1.4. Chemical Admixtures

For the reference ready-mix concrete mixtures, a high-performance superplasticizer (Admixture 1) based on PC(E) technology was employed. In the case of recycled aggregate concrete mixtures, various trial mixes were conducted using specialized admixtures from different manufacturers. These admixtures were designed primarily to mitigate the water absorption of the aggregate, thereby influencing the workability and setting time of the fresh concrete. Admixture 2, a Generation I plasticizing admixture, was utilized to address the high water absorption of recycled aggregate and the related issues of consistency and initial setting time due to its anti-adsorption properties. It was paired with Admixture 3, a Generation IV PC(E) acrylate-type superplasticizer from the same manufacturer. The dosage of admixtures was determined to achieve the same initial consistency on the basis of water uptake through trial mixing and observations during the mixing process. Special attention was given to prevent bleeding, postfluxing, and premature consistency loss while adjusting the admixture dosage. A standard admixture was used for the reference concrete (REF-1) for the given cement type, as it did not contain recycled aggregate. As the R0, R1, R2, and R3 concrete mixes were made at different times with aggregates from different sources, we tested several types of admixtures from several manufacturers on the mixes to find the most suitable solution.

#### 2.1.5. Mixing Water

Drinking-quality tap water was used for all the mixtures.

### 2.2. Mix Designs

The volume of the different aggregate fractions was the same in all the mixtures: the 0/4 fraction was 40%, the 4/8 fraction was 25%, and the 8/16 fraction was 35%. The type and volume of the recycled aggregate in the mixtures varied with respect to the coarse fraction (Table 4).

The Hungarian standard MSZ 4798:2016 [45] also includes a water resistance exposure class, which is denoted XV2(H). The design of the reference mix for ready-mix concrete (REF-1) was based on a designed water content of 170 L/m^3^. The cement content in the mix was chosen so that the water–cement ratio of the concrete designed with these parameters would meet the requirements of the current environmental exposure class XV2 (H). [MSZ 4798:2016 NAD F1 Table]. With the recycled aggregates, 12 different mixtures were prepared, as shown in Table 5.

The type of recycled aggregates, the amount added, and whether the aggregates were added wet or dry differed between the mixtures. Notably, the initial setting time of the REC-1 and REC-2 mixes, which utilized recycled aggregate R0, fell significantly below that of the reference mixes, with the concrete becoming unworkable after approximately 20 min. In light of this issue, modifications to the original recycled aggregate formulations were necessary. The two modified recycled concrete mixes, REC-3 (15 m) and REC-4 (30 m), were developed. The primary challenge with the formulation of the REC-1 (15) and REC-2 (30) mixtures, concerning setting time and workability, is attributed to the amount of water absorbed by the mixing water and the additives.

### 2.3. Concrete Testing Methods

Testing was conducted on both fresh and hardened concrete, as well as on the selected recycled aggregates, with a focus on designing mixes suitable for everyday construction.

(1) Aggregates:

In addition to particle size distribution, the recycled aggregates were tested for micro-Deval (EN 1097-1:2012) [46] and Los Angeles values (EN 1097-2:2020) [47], water absorption, and density (according to EN 1097-6:2022, clause 8, using a pycnometer method) [48];

(2) Concrete mixing procedure and testing methods:

One of the main objectives of our study was to improve the initial setting time of recycled concrete. To assess the consistency of the ready-mix concrete, we measured the area under the curve using a flow table to test the MSZ EN 12350-5:2019 [49] standard at 5, 30, 60, and 90 min. The mixed concretes were prepared using a Beckel Eimermischer type 8-L mixer (Schwallungen, Germany). For mixes intended for fresh and hardened concrete tests, a Schwelm Zyklos ZK75HE0-type vertical axis mixer (Rohlbach, Germany) was used. The reason for this was the limited supply of raw materials, as our aim was first to select the right admixture and define its appropriate dosage rate and then to prepare mixtures for tests on fresh and hardened concrete samples. Only initial setting time was tested on the mixes prepared in the smaller mixer, whereas the tests on the air content and other fresh and hardened concrete properties were carried out on samples of mixes prepared in the large mixer.

For mixtures prepared under both a saturated surface that was dried and pre-soaked conditions, the concrete mixing time was adjusted to 1 min after dry mixing, followed by the addition of water and admixtures to achieve homogeneity.

In cases where recycled admixtures were added in a dehydrated state only, dry mixing was omitted. Instead, the special flow additive was gradually incorporated with 2/3 of the mixing water, following the manufacturer’s instructions. The duration of homogenization was approximately 60 s. The wet mixture then underwent further mixing for 2 min, during which the remaining mixing water and superplasticizer were gradually added to achieve the desired workability. Following the mixing process, the properties of the fresh concrete were tested. Additionally, the fresh concrete properties (consistency, air content, and bulk density) of the reference mixes (REF-1) were compared with those of the mixes prepared with recycled aggregates from different sources added at 15% and 30%;

(3) Hardened concrete properties and testing methods:

Compressive strength and water absorption tests were conducted in accordance with EN 12390-3:2019 [50] and EN 12390-8:2019 [51], respectively, at 7 and 28 days. For compressive strength testing, concrete samples were prepared and stored in a mixed medium (immersed in water for 7 days, followed by storage in laboratory air) according to MSZ 4798 [46]. For water resistance testing, the samples were submerged in water according to EN 12390-2:2019 [52]. Water resistance testing was executed following MSZ EN 12390-8:2019 [52] standards. As per MSZ 4798:2016/2M:2018 [53], the water resistance test aimed to achieve a maximum water penetration depth XV2(H) of 35 mm for each test sample.

## 3. Results and Discussions

### 3.1. Recycled Aggregates Testing

Table 6 shows the mechanical and physical properties of the recycled aggregates.

According to the table below, the aggregate with the highest water absorption among the different aggregates is type R3 (mixed demolition waste), which includes construction ceramics, brick rubble, and other pavements. The aggregates of the reclaimed precast panels (type R1) had exceptionally high porosity. Contrary to expectations, this aggregate did not perform best in terms of crushing and abrasion resistance, which was achieved by the pure crushed concrete aggregate (type R2). We intended to verify both the strength and the environmental exposure class requirements by measuring the properties of the setting time.

**Figure 2:**
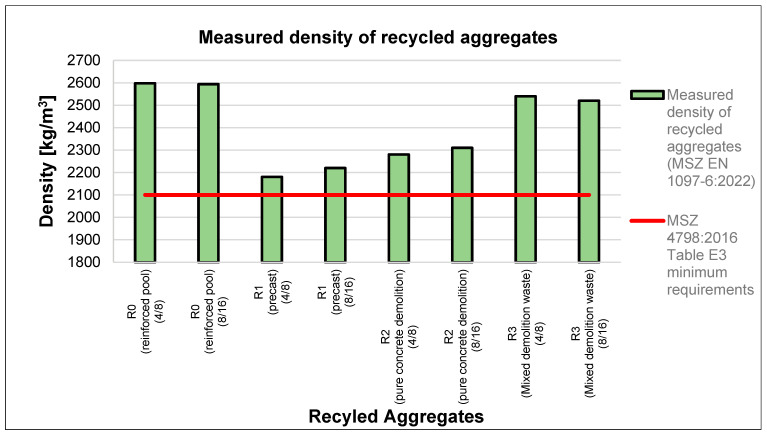
Measured density of recycled aggregates.

**Figure 3:**
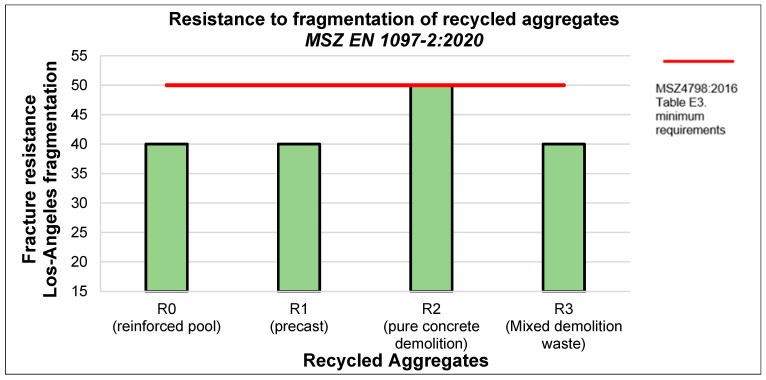
Resistance to fragmentation of recycled aggregates.

The bulk density of recycled aggregate is approximately 15–20% lower for types R1 and R2 and 3–7% lower for aggregates from precast slabs (type R3) and demolished reinforced concrete swimming pools (type R0) than for natural aggregates. This disparity can be attributed to the presence of cement pebbles, which are more porous than naturally graded gravel [Figure 2]. However, the results depicted in the graph below indicate that the recycled aggregates did not meet the minimum requirements outlined in EN 206 [54] [Figure 3].

### 3.2. Fresh Concrete Properties and Testing Methods: Initial Setting Time

**Figure 4:**
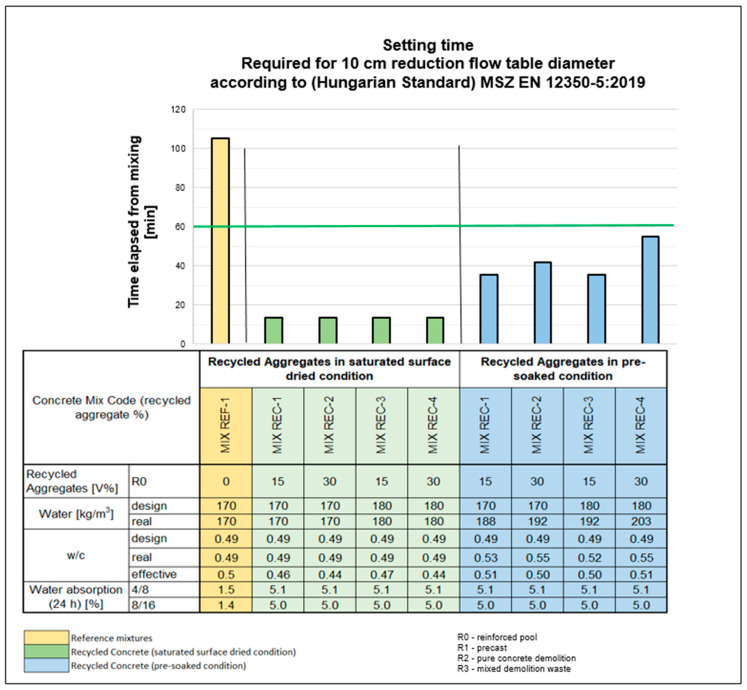
Changes in the initial setting time during the dosing of recycled aggregates under saturated surface-dried (SSD) and presoaked conditions.

Consistency testing was conducted at 5, 30, 60, 90, and 120 min, if still measurable, to assess the behavior of the mixtures over time. The initial target consistency was defined as an area value of 540 mm. The REC-1 to REC-4 mixtures were prepared using two methods. Initially, the aggregates were incorporated into the mixture under air-dry conditions. However, as indicated in Section 2.2, the setting time of these dry-added mixtures fell short of expectations, even with increased mixing water and additional additives. Additionally, the secondary aggregates, dosed at the same rate, absorbed moisture from the mixing water, further affecting the performance of the mixtures. Consequently, the REC-1 to REC-4 mixtures were also produced by soaking the recycled aggregates. While soaking positively influenced the initial setting time, the mixtures remained unsuitable for industrial use [Figure 4]. Building on these findings, further trial mixes were conducted using aggregates from various sources, primarily incorporating water absorption-compensating additives (REC-7 to REC-12). The dosage of additives was determined based on the results of preliminary test mixes and water absorption data.

**Figure 5:**
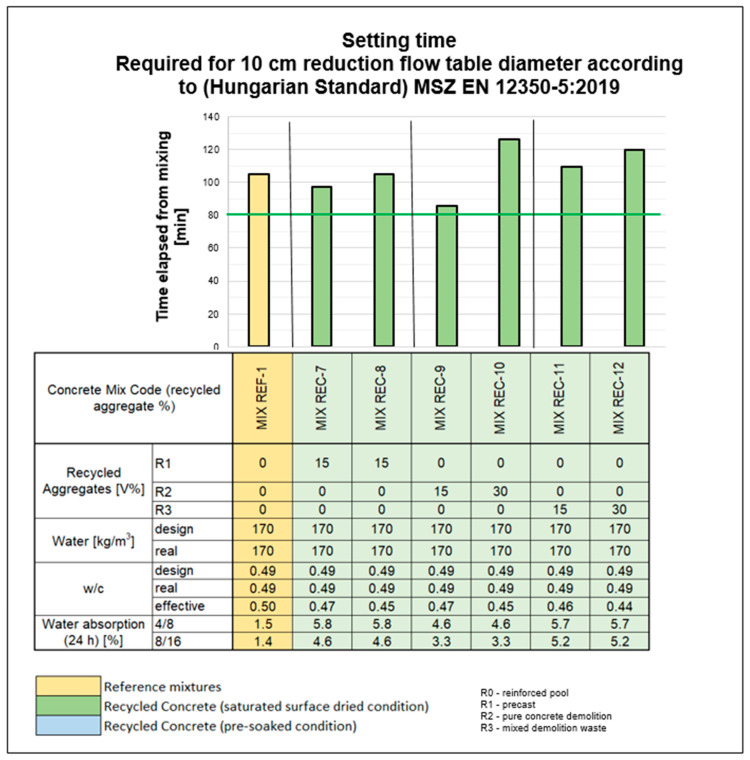
Variations in the initial setting time.

The addition of the water-reducing additive and flow agent provided the expected consistency for mixtures with 15% substitution (REC-7 and REC-9), which remained workable even after 1.5 h. However, as the substitution percentage increased to 30%, the dosage of the special flow agent was also increased compared with that of the 15% substitution mixture to maintain the desired initial consistency. This adjustment was necessary due to the expected decrease in workability and shelf life with higher substitution percentages, which was attributed to increased water absorption by the recycled aggregates. Consequently, varying and sometimes higher additive dosages were required [Figure 5]. It was demonstrated that shelf life is not improved by increasing the amount of mixed water by the amount absorbed by the recycled aggregate if the recycled aggregate is added to the mixture in a dry state. In summary, for mixtures with high porosity and substitution rates, simply adding a superplasticizer after initial water absorption does not effectively address consistency and shelf-life issues if the absorption is not mitigated from the outset.

### 3.3. Tests on Fresh Concrete: Consistency, Air Content, and Density

#### Determination of Fresh Concrete Density According to EN 12350-6:2019 [55] and Air Content According to EN 12350-7:2019 [56]

**Figure 6:**
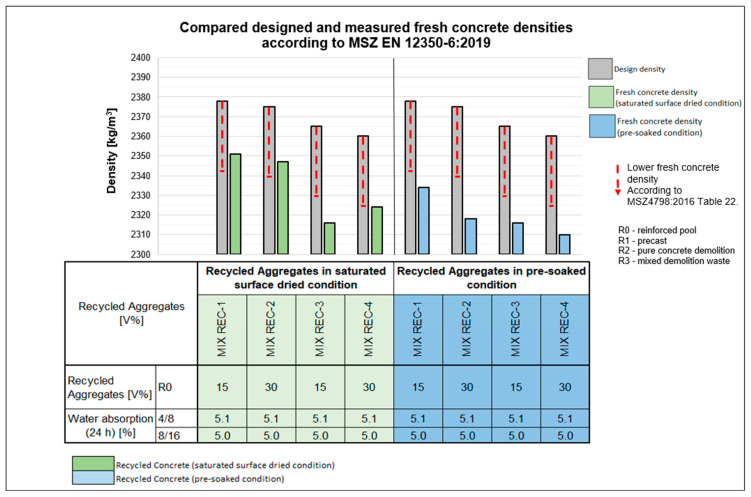

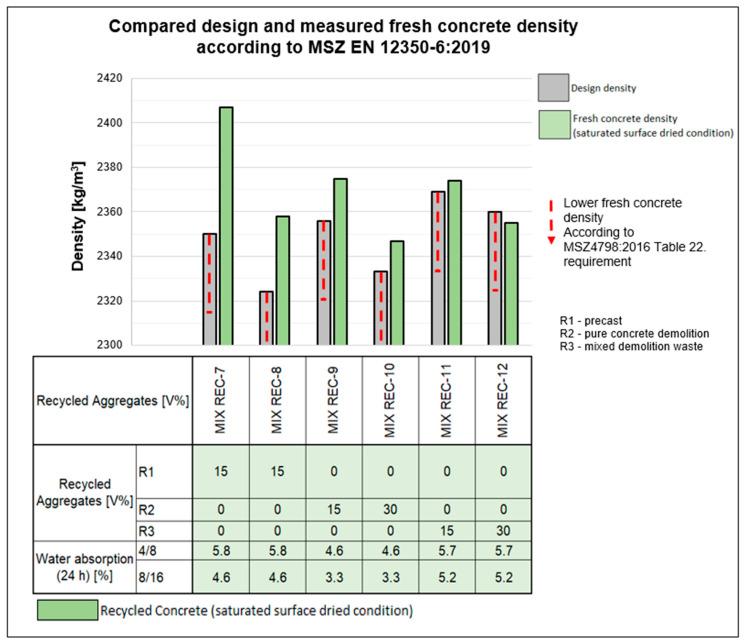
Comparison of designed and measured fresh concrete densities.

Figure 6 shows the density of the designed and fresh concrete for various mixtures. Our findings indicate that, without suitable admixtures, the targeted limits of the designed fresh concrete densities could not be met. However, when the mixtures included a special admixture for recycled aggregate, the densities remained within the specified limits.

### 3.4. Tests on Hardened Concrete [57,58]

#### 3.4.1. Compressive Strength 

**Figure 7:**
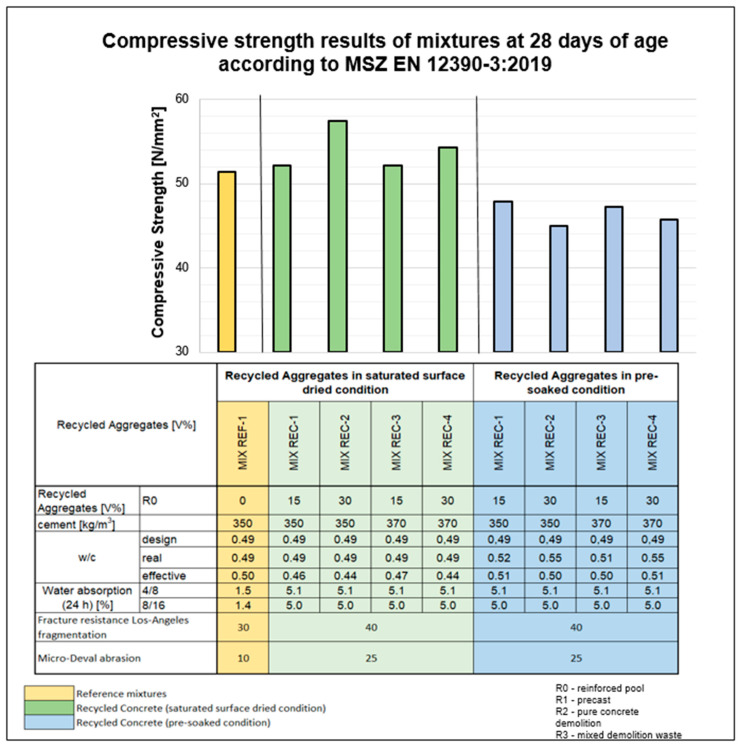
Compressive strength results of the mixtures at 28 days (saturated surface-dried (SSD) and pre-soaked condition of aggregates).

**Figure 8:**
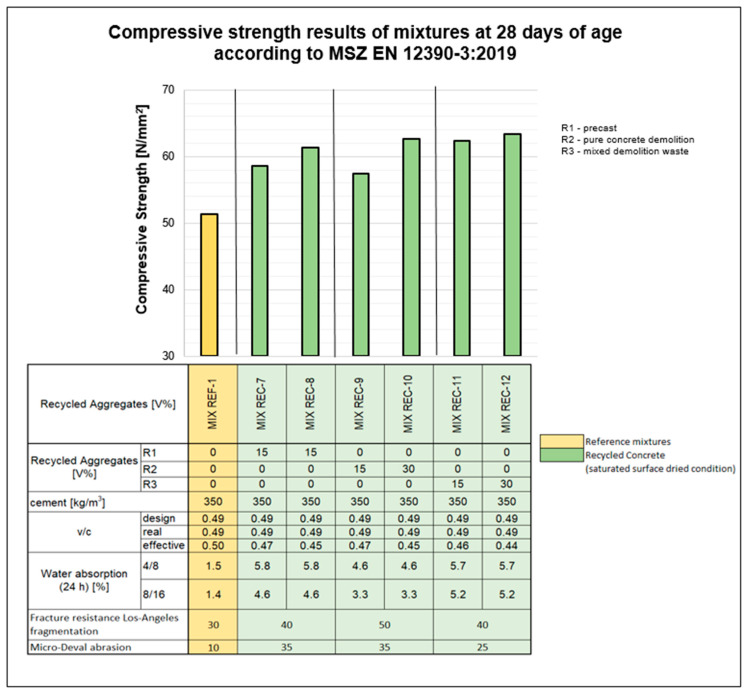
Average strength of the mixtures at 28 days.

The compressive strength results of the samples are summarized in Figure 7 and Figure 8.

Figure 7 shows that, compared with the reference mix, incorporating recycled aggregate in the saturated surface-dried state enhances the compressive strength of the concrete at 28 days. This improvement in compressive strength is also noticeable when the proportion of recycled aggregate in the coarse fraction increases from 15 to 30%. This phenomenon is likely due to the recycled aggregate absorbing moisture from the concrete, thereby reducing the effective water–cement ratio and limiting the formation of capillary pores. Conversely, pre-soaking the aggregate reduces the compressive strength of the concrete compared with that of the reference mixture, possibly because the absorbed water increases the capillary pore volume. Our tests indicate that, when the recycled aggregate is combined with a specially developed additive, the compressive strength further increases with the increasing recycled aggregate content compared with that of the reference concrete (see Figure 8). The effective water–cement ratio is calculated as the ratio of the water in the design mixture minus the water absorbed by the aggregate to the amount of cement. This value is inherently lower than the base (design) water–cement ratio.

#### 3.4.2. Water Resistance Test

**Figure 9:**
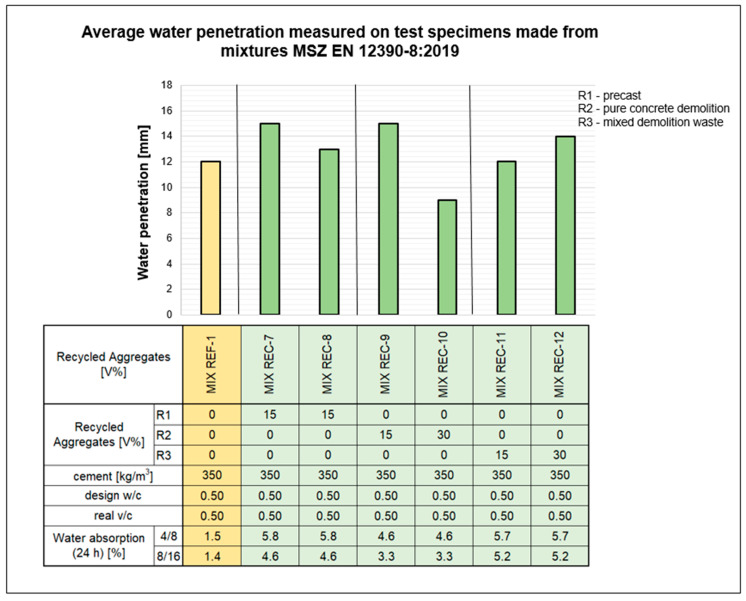
Average water penetration measured on test samples made from mixtures.

Figure 9 shows the average values of the water penetration depths of the mixtures measured during the water resistance test. At this stage, the different mixtures all meet the required values for the environmental classes XV2 (H) and even XV3 (H). The water resistance of concrete is affected by the type of recycled aggregate. When pure crushed concrete was used as a recycled aggregate, a 15% dosage significantly enhanced water impermeability, but at a 30% dosage, this effect was potentially offset by the denser structure. Conversely, with mixed debris, the higher water absorption of the aggregate led to increased deterioration when the dosage was increased from 15 to 30%.

## 4. Conclusions

The findings indicate that an optimized water–cement ratio and the use of specialized additives significantly enhance RCA concrete performance. Specifically, the 30% RCA mixture (REC-10) achieved a 28-day compressive strength of 62.7 MPa, exceeding both the reference concrete (REF-1) at 51.4 MPa and the 15% RCA mixture (REC-9) at 58.2 MPa. The improvement is attributed to the optimized mix design, which counteracts the higher porosity and water absorption of RCA. However, RCA from mixed demolition waste (REC-12) performed slightly worse, achieving 55.6 MPa, highlighting the variability in RCA quality.

Water resistance tests further demonstrated the benefits of optimized RCA mixtures. The maximum water penetration depth for REF-1 was 15 mm, whereas REC-10 (30% RCA) exhibited only 11 mm, indicating enhanced water impermeability. Similarly, the REC-9 mixture (15% RCA) achieved 12 mm, while REC-12 (from mixed demolition waste) showed a higher penetration depth of 16 mm, reflecting the influence of RCA composition. These results suggest that carefully selected RCA can enhance water resistance, but quality control remains crucial. In terms of fresh concrete properties, RCA mixtures initially showed reduced workability and rapid setting due to high water absorption.

Without admixtures, RCA concrete became unworkable within 20 min. However, the use of specialized water-reducing additives extended the initial setting time to 80 min, ensuring practical usability. Additionally, the bulk density of RCA concrete was approximately 3–7% lower than that of natural aggregate concrete, correlating with RCA’s higher porosity and water absorption, which ranged from 3.3 to 5.8% depending on the source material.

(i) Laboratory tests conducted on crushed recycled aggregates confirmed that concrete incorporating these aggregates met the compressive strength and water resistance standards despite the recycled aggregates themselves not meeting all standard requirements. These findings suggest that recycled aggregates can serve as a feasible substitute for natural coarse aggregates in various concrete compositions. However, it is crucial to emphasize that each batch of crushed aggregate is unique and requires thorough testing and evaluation, including trial mixing, for suitability in specific applications;
(ii) According to the experimental results, the concrete mixtures with 15 and 30% recycled aggregates presented a higher 28-day compressive strength compared to the conventional reference mixes with gravel aggregates. The addition of the recycled aggregate in a saturated, surface-dried state increased the 28-day compressive strength of the concrete, and a further increase was observed when the recycled aggregate ratio was increased from 15% to 30%. This phenomenon can be attributed to the favorable water–cement ratio. As the recycled aggregate absorbs moisture from the concrete, the overall water–cement ratio decreases, resulting in reduced capillary pore formation;

In contrast, for pre-soaked aggregate, increasing the proportion of recycled coarse aggregate in the concrete led to a decrease in compressive strength compared to the reference concrete, probably because the additional water in the aggregate increased the capillary pore system. Tests have also shown that, when the recycled aggregate was combined with a specially formulated aggregate, the compressive strength increased, even with a higher recycled aggregate content, compared with that of the reference concrete.

(iii) Experiments demonstrated that the loss of initial setting time could not be compensated for by increasing the amount of mixing water when the recycled aggregate was added to the mixture in a dehydrated state. For mixtures with high porosity and high substitution rates, if the initial water uptake is not eliminated (adsorption inhibition) upon the first water contact, the subsequent addition of the superplasticizer does not solve the consistency and shelf-life problems. Additionally, the varying composition of the crushed recycled aggregates could influence the effectiveness of both the mixing water and the additive;
(iv) The results of the tests demonstrated that all of the mixtures met the maximum water penetration requirement for environmental class XV2 (H) according to the Hungarian standard MSZ 4798:2016/2M [53]. This standard specifies a maximum permissible individual depth of water penetration of 35 mm. It should be noted that, while the relevant standard does not recommend the use of recycled aggregate in waterproofing structures, our research suggests that further testing and potential revisions to regulations may be warranted.

## Figures and Tables

**Table 1:** Summary of previous research on concrete compressive strength.

Reference	RCA Content (%)	Key Findings on Compressive Strength	Type of RCA Used	Compressive Strength Class (MPa)	Additional Notes
Fong et al. [1,2]	30%	Demonstrated feasibility of using RAC for structural applications	Crushed concrete	30–40	Used in Hong Kong infrastructure projects
Lim et al. [3]	50%	Reduced workability, lower strength	Mixed RCA	45–52	-
Ramakrishnaiah et al. [4]		Investigated effects of polypropylene fibers on geopolymer concrete strength	Fly ash-based recycled aggregate	25–35	Enhanced flexural strength observed
Ismail and Ramli [5,6]	30%	Pre-treatment improved strength	Pre-treated RCA	43–47	Acid treatment applied
Tiznobaik et al. [7]	30%	Found that RCA can achieve C20/25 and C30/37 strength classes	Crushed demolition waste	25–37	Long-term durability assessed
Zhang et al. [8]	20%	Studied surface modifications to improve compressive strength	Coarse recycled aggregates	35–45	Modified surfaces improved bond strength. Comparable to natural aggregate concrete.
Ju et al. [9]	50%	Strength reduction due to higher porosity	Mixed RCA	35	-
Sampaio et al. [10]	35%	Evaluated RAC in high-performance concrete (C50/60)	Mixed recycled aggregates	50–60	Suitable for structural applications
Robalo et al. [11]	20%	Strength enhancement with specific additives	Recycled coarse aggregates	50	Additives improved bonding

**Table 2:** Summary of previous research on setting time.

Reference	RCA Content (%)	Findings on Setting Time	Type of RCA Used	Initial Setting Time (min)	Final Setting Time (min)
Ismail & Ramli [5,6]	30%	Surface treatment with acid improved setting time consistency	Acid-treated RCA	45	120
Aili et al. [12]	30%	Reported that higher porosity in RCA led to increased setting time variability	Untreated RCA	60	150
García-González et al. [13]	30%	Pre-saturation technique enhanced workability but slightly increased setting time	Pre-saturated RCA	50	130
Ju et al. [9]	50%	CO_2_ curing accelerated setting time improvements	Carbonated RCA	40	110
Wang et al. [14]	35%	Developed a numerical model for setting time estimation in RAC	Various recycled aggregates	55	140
Babraddin et al. [15]	40%	Higher RCA led to shorter setting time	Mixed RCA	32	110

**Table 3:** Summary of previous research on concrete water resistance.

Reference	RCA Content (%)	Key Findings on Impermeability	Type of RCA Used	Water Penetration Depth (mm)	Additional Notes
Ahmad SI [16]	30%	Found that RAC had significantly higher permeability (225–550%) than NAC	Crushed clay brick as coarse aggregate	15–35	Increased porosity reduced resistance
Robalo et al. [11]	30%	Reported that RCA increases porosity and water absorption	High-paste RCA	20–40	High absorption affected durability
Skocek et al. [17]	40%	Developed a method to reduce RCA porosity through controlled separation	Recycled sand and coarse aggregates	10–25	Improved compaction reduced voids
Al-Kheetan et al. [18]	50%	Use of nano-ZnO improved impermeability and reduced pore size	Nano-particle-treated RCA	8–20	Nano-modifications reduced permeability
Zhong et al. [19]	20%	Evaluated water absorption changes due to freeze-thaw cycles in RAC	Freeze–thaw modified RCA	12–30	Variable results based on exposure conditions

**Table 4:** Coarse aggregate content in each mixture.

Concrete Mix Code	Coarse Aggregate		
	Natural	Recycled	Recycled Material Source
MIX REF-1	100%	0%	
MIX REC-1 (15)	85%	15%	reinforced pool: R0
MIX REC-2 (30)	70%	30%	reinforced pool: R0
MIX REC-3 (15 m)	85%	15%	reinforced pool: R0
MIX REC-4 (30 m)	70%	30%	reinforced pool: R0
MIX REC-7 (15)	85%	15%	precast: R1
MIX REC-8 (30)	70%	30%	precast: R1
MIX REC-9 (15)	85%	15%	pure concrete demolition: R2
MIX REC-10 (30)	70%	30%	pure concrete demolition: R2
MIX REC-11 (15)	85%	15%	mixed demolition waste: R3
MIX REC-12 (30)	70%	30%	mixed demolition waste: R3

Reference concrete mark: REF-1: C30/37-XC3-XV2 (H)-16-F4.

**Table 5:** Concrete mix proportions.

Concrete Mix Code	Cement [kg]	Water [kg]	W/C	Sand 0/4 [kg]	Coarse Aggregate [kg/m^3^]					Admixture [mc%]			Design Density [kg/m^3^]
					NA 4/8; 8/16	RAC 4/8; 8/16				Admixture 1	Admixture 2	Admixture 3	
					N	R0	R1	R2	R3				
MIX REF-1	350	170	0.49	744	1116	0	0	0	0	0.70			2383
MIX REC-1 (15)	350	170	0.49	742	944	167	0	0	0	0.50			2378
MIX REC-2 (30)	350	170	0.49	742	774	335	0	0	0	0.50			2375
MIX REC-3 (15 m)	370	180	0.49	761	892	157	0	0	0	0.50			2365
MIX REC-4 (30 m)	370	180	0.49	760	731	103	0	0	0	0.55			2360
MIX REC-7 (15)	350	170	0.49	741	949	0	137	0	0		0.45	0.70	2350
MIX REC-8 (30)	350	170	0.49	739	783	0	275	0	0		0.70	1.00	2324
MIX REC-9 (15)	350	170	0.49	741	949	0	0	142	0		0.45	0.65	2356
MIX REC-10 (30)	350	170	0.49	740	781	0	0	287	0		0.70	0.85	2333
MIX REC-11 (15)	350	170	0.49	740	947	0	0	0	156		0.60	0.85	2369
MIX REC-12 (30)	350	170	0.49	739	779	0	0	0	316		0.85	1.10	2360

**Table 6:** Natural and recycled aggregate properties.

Natural/Recycled Aggregates Size [mm]	Water Absorption (24 h) [%]	Los Angeles	Micro-Deval	Density [Mg/m^3^]
Standard	EN 1097-6	EN 1097-2	EN 1097-1	EN 1097-6
4/8				
NA	1.5			2.63
R0	5.1			2.63
R1	5.8			2.18
R2	4.6			2.28
R3	5.7			2.54
8/16				
NA	1.4			2.68
R0	5.0			2.58
R1	4.6			2.22
R2	3.3			2.31
R3	5.2			2.52
NA		<30	<10	
R0		39	27	
R1		39	22	
R2		41	31	
R3		38	24	

## Data Availability

The original contributions presented in the study are included in the article, further inquiries can be directed to the corresponding author.
